# PML Is Limiting NLRP3 Inflammasome Activity in Human Endothelial Cells

**DOI:** 10.3390/cells14241961

**Published:** 2025-12-10

**Authors:** Celine Huajia Liem, Gustav Steinemann, Nona Ghiroltean, Yvonne Yvonne, Hana Sakr, Huyen Nguyen, Oliver Baum, Janine Berkholz

**Affiliations:** 1 Vascular Biology Research Group, Institute of Physiology, Charité–Universitätsmedizin Berlin, 10117 Berlin, Germany; celine.liem@charite.de (C.H.L.); gustav.steinemann@charite.de (G.S.); nona.ghiroltean@charite.de (N.G.); yvonne.yvonne@charite.de (Y.Y.); hana.sakr@charite.de (H.S.); huyen.nguyen@charite.de (H.N.); oliver.baum@charite.de (O.B.); 2 German Centre for Cardiovascular Research (DZHK), 13353 Berlin, Germany

**Keywords:** PML, NLRP3, inflammasome, endothelial cell

## Abstract

NLRP3 inflammasomes are transient large protein aggregates involved in the regulation of the innate immune response but are also associated with endothelial dysfunction during vascular inflammation. While NLRP3 inflammasome assembly and activation is well characterized in immune cells, its role in endothelial cell function remains incompletely understood. This study analyses the function of promyelocytic leukemia (PML) protein, a nuclear scaffold protein that forms so-called PML nuclear bodies (PML-NBs), in the regulation of NLRP3 inflammasome activation in endothelial cell cultures. Following LPS priming and subsequent ATP-induced activation, PML played a dual role: 1. It enhanced NF-kB-dependent transcription of inflammasome components (NLRP3, pro-caspase-1 and pro-IL-1β). 2. At the same time, a post-translational reduction in NLRP3 protein levels and reduced ASC oligomerization were observed, leading to impaired inflammasome activation, as evidenced by lower caspase-1 activity and reduced IL-1β secretion. Proper formation of PML-NBs was critical for this regulatory effect on NLRP3 inflammasome formation, as PML-NBs retained ASC in the nucleus and post-translationally modified NLRP3, presumably affecting its stability. Taken together, these findings suggest that PML represents a regulatory checkpoint in endothelial inflammasome activation, preventing excessive inflammatory responses that could contribute to vascular dysfunction associated with chronic inflammation.

## 1. Introduction

NLRP3 inflammasomes are transient multiprotein complexes in the cytosol that play a central role in innate immunity by sensing cellular stress and microbial infections [1]. They consist of three key components [2]: (1) NLRP3 (NOD-like receptor pyrin domain-containing 3), a sensor protein of the NOD-like receptor family; (2) ASC (apoptosis-associated speck-like protein containing a CARD), an adaptor protein that links oligomerized NLRP3 molecules and recruits pro-caspase-1 via its pyrin (PYD) and caspase activation and recruitment domains (CARD); and (3) pro-caspase-1, an inactive precursor that, upon recruitment by ASC, undergoes autoproteolytic cleavage to form active caspase-1.

NLRP3 inflammasome activation requires two temporarily successive processes [3]: The first step, priming, involves the upregulation of transcription of the inflammasome components, while the second step, activation, refers to the translation and subsequent assembly of the inflammasome proteins. During the priming phase, extracellular signaling molecules, such as cytokines or pathogen-associated molecular patterns (PAMPs, such as LPS, viral RNA, and bacterial toxins) trigger the intracellular NF-κB signaling cascade, leading to the induction of NLRP3, pro-IL-1β, and pro-IL-18 mRNA [2,3]. In the activation phase, PAMPs or damage-associated molecular patterns (DAMPs), such as heat shock proteins, extracellular DNA, and purine metabolites like ATP and ADP, initiate signaling cascades that cause potassium efflux, shifts in intracellular calcium ion-levels, and higher production of mitochondrial reactive oxygen species (ROS) which then promote NLRP3 oligomerization and inflammasome assembly [2,3]. A key mechanism of NLRP3 activation involves ATP release from damaged cells, which stimulates P2X7 receptor signaling, leading to potassium ion efflux—this is a crucial step for inflammasome activation [3,4]. As a consequence of activation, NLRP3 inflammasomes upregulate signaling pathways that trigger an inflammatory response, with the proinflammatory cytokines IL-1β and IL-18 being processed by activated caspase-1 from their precursor forms (pro-IL-1β and pro-IL-18) into their secreted, bioactive forms [2,3].

Although this inflammasome activation is predominantly observed in immune cells such as macrophages and dendritic cells [5], it also occurs in endothelial cells (ECs) [6,7,8,9]. Like immune cells, ECs also express pattern-recognition receptors (PRRs) that bind PAMPs and DAMPs [10]. The activation of the NLRP3 inflammasome in ECs leads to an increased expression of proinflammatory mediators, including IL-6, C-reactive protein (CRP), adhesion molecules, and chemokines [6,11]. This secretion activity promotes leukocyte recruitment and adhesion, contributing to the amplification of inflammatory signaling cascades. Additionally, NLRP3 inflammasome activation disrupts EC junctions—particularly VE-cadherins—thereby increasing endothelial permeability [7]. This weakening of the endothelial barrier subsequently promotes vascular leakage and impaired homeostasis, which exacerbate both inflammatory and cardiovascular pathology [6]. In summary, NLRP3 activation in ECs, contributes significantly to vascular inflammation and endothelial dysfunction, playing a crucial role in the progression of cardiovascular and inflammatory diseases such as atherosclerosis.

Emerging evidence highlights the critical role of the promyelocytic leukemia (PML) protein in regulating inflammatory responses, particularly in immune cells and ECs [12,13,14,15,16,17,18]. Upon activation, PML proteins form PML nuclear bodies (PML-NBs), which are dynamic nuclear multiprotein complexes involved in many cellular processes such as apoptosis, senescence, and antiviral responses [19]. The assembly of PML-NBs is dependent on PML itself and requires SUMOylation [20,21]. PML-NBs exhibit various cellular functions through different mechanisms, including: (1) serving as protein reservoirs, (2) acting as hubs of nuclear activity, (3) playing a role in RNA regulation, and (4) functioning as hotspots for post-translational protein modifications such as SUMOylation, phosphorylation, and ubiquitination [16]. Alternative splicing generates at least 12 PML isoforms with distinct subcellular localizations, including nuclear (PML1-6) and cytoplasmic variants (PML1,7) [22] with isoform 4 being one of the best characterized isoforms to date. Notably, PML1 shuttles between the nucleus and cytoplasm, suggesting a dynamic regulatory role of this PML form. Beyond its established functions in tumor suppression and apoptosis, PML proteins are increasingly recognized as modulators of inflammation, particularly in viral infections and innate immunity [16,17,23,24]. Its expression is elevated in inflamed tissues and tightly regulated by cytokines [25].

In this study, we validated the hypothesis that PML plays a central role in inflammatory signaling pathways associated with NLRP3 activation in ECs. We found that, on the one hand, PML increases the inflammatory susceptibility of ECs after exposure to PAMPs and DAMPs by increasing the expression of specific inflammasome components in an NF-kB-dependent manner. On the other hand, the data show that PML-NBs may act as a regulatory mechanism that limits excessive inflammatory responses by restricting inflammasome assembly. This newly identified function of PML and PML-NBs in ECs provides valuable insights into endothelial inflammatory processes, which are crucial for the understanding of vascular diseases.

## 2. Materials and Methods

### 2.1. Cell Culture

EA.hy926 cells (Elabscience, Housten, TX, USA) were cultured in Dulbecco’s Modified Eagle’s Medium (DMEM) and the human myeloid THP-1 cell line (DSMZ, Braunschweig, Germany) in RPMI 1640, both supplemented with 10% fetal bovine serum (FBS; Gibco, Thermo Fisher Scientific, Waltham, MA, USA). Human umbilical vein endothelial cells (HUVECs) were isolated as previously described [26] and cultured in Endothelial Cell Growth Medium (PromoCell, Heidelberg, Germany). All cell cultures were kept at 37 °C in a humidified incubator with 5% CO_2_. The isolation of HUVECs was ethically authorized by the Ethics Committee of Charité–Universitätsmedizin Berlin, Germany (EA4/107/17). Umbilical cords were collected from healthy donors after obtaining written informed consent according to the Declaration of Helsinki.

### 2.2. Cell Stimulation

EA.hy926 cells were seeded in appropriate culture wells and allowed to reach 70–80% confluency prior to stimulation. For inflammasome activation, cells were primed with lipopolysaccharide (LPS, 1 µg/mL; Escherichia coli O55:B5, Sigma-Aldrich, St. Louis, MO, USA) for 24 h in complete medium to induce priming. To activate the NLRP3 inflammasome, ATP (5 mM; Thermo Fisher Scientific, Waltham, MA, USA) was added during the final 60 min of LPS stimulation. To induce AIM2 inflammasome activation, EA.hy926 cells were transfected with poly(dA:dT) (5 µg/mL; Cell Signaling Technology, Danvers, MA, USA) for 8 h. Where indicated, cells were transfected with the respective expression vectors prior to stimulation/transfection and subsequently cultured under the same conditions.

### 2.3. Treatment of Cells with PDTC or Bay 11-7082

Pyrrolidine dithiocarbamate (PDTC; 10 μM; Roth, Karlsruhe, Germany) and Bay 11-7082 (10 μM; Calbiochem, San Diego, CA, USA), both dissolved in dimethyl sulfoxide (DMSO), were added to the medium, with DMSO alone serving as the negative control.

### 2.4. Treatment of Cells with MG132 or Cycloheximide

MG132 (10 μM; Sigma-Aldrich) and cycloheximide (10 µg/mL; Roth), both prepared in DMSO, were applied to the medium. DMSO alone was used as the negative control. For proteasome inhibition, MG132 was added 30 min prior to ATP stimulation. For protein stability analysis, cycloheximide was added simultaneously with ATP stimulation (CHX chase), and cells were harvested at indicated time points.

### 2.5. Transfection with PML Expression Vector

EA.hy926 cells were transiently transfected with expression plasmids as previously described [27]. Briefly, cells received pEGFP-C1-PML-IV (PML-IV) [28], pEGFP-C1-PML-V (PML-V) [28], or PML-3/pSG5 (PML-IV-Mut) [21] using TurboFect (Thermo Fisher Scientific, USA) according to the manufacturer’s instructions. For SUMO-related analyses, pcDNA3-Sumo1-HA was used. Transfection efficiency was evaluated by RT-qPCR and immunoblotting. The pEGFP-C1-PML-IV and pEGFP-C1-PML-V plasmids were kindly provided by PD Dr. Peter Hemmerich (Leibniz Institute for Ageing Research—Fritz Lipmann Institute, Jena, Germany), pcDNA3-Sumo1-HA by Prof. Hans Will (Hamburg, Germany), and PML-3/pSG5 by Pier Pandolfi (Addgene plasmid # 50939; http://n2t.net/addgene:50939; RRID: Addgene_50939; accessed on 1 September 2025). “Control cells” refer to cells transfected with the respective empty vector.

### 2.6. Transfection with Small Interfering RNA (siRNA)

EA.hy926 cells were electroporated with a pool of four unrelated siRNAs targeting PML (25 nM each; Dharmacon, Lafayette, CO, USA). A non-targeting “scrambled” siRNA served as the negative control. Knockdown efficiency was evaluated 24 h post transfection by RT-qPCR and/or immunoblotting.

### 2.7. RNA Isolation, Reverse Transcription, and Real-Time PCR

Total RNA was extracted from EA.hy926, HUVECs, and THP-1 cells using the GeneMATRIX Universal RNA Purification Kit (EURx, Gdańsk, Poland) according to the manufacturer’s instructions. Semi-quantitative RT-qPCR was performed on a QuantStudio 5 system (Applied Biosystems, Foster City, CA, USA) using GoTaq qPCR Master Mix (Promega, Madison, WI, USA). Primer sequences, product sizes, and annealing temperatures are listed in Table 1 (primers from Eurofins, Ebersberg, Germany). Melting curve analysis confirmed single-transcript amplification. Relative mRNA levels were calculated using the comparative CT (2^−ΔΔCT^) method with GAPDH as the reference, and non-RT and non-template controls were included in all reactions.

### 2.8. Immunoblotting

Cell lysates were prepared by homogenizing cells in RIPA buffer (Santa Cruz, Dallas, TX, USA) supplemented with protease inhibitors and 20 mM N-ethylmaleimide (both Sigma-Aldrich, Darmstadt, Germany) for 15 min at 4 °C, followed by centrifugation. Protein concentrations were determined using the BCA Protein Assay Kit (Thermo Fisher Scientific, USA). Equal amounts of protein (20 µg) were separated by SDS–PAGE and transferred to nitrocellulose membranes (GE Healthcare, Chicago, IL, USA). Cytoplasmic and nuclear fractions were obtained using the NE-PER Nuclear and Cytoplasmic Extraction Kit (Thermo Fisher Scientific, USA) according to the manufacturer’s instructions. Primary antibodies were used at the following dilutions: anti-PML (1:1000, Novus Biologicals, Centennial, CO, USA), anti-SUMO-1 (1:500, Elabscience), anti-NLRP3 (1:1000, Proteintech, Rosemont, IL, USA), anti-ASC (1:1000, Cell Signaling), anti-Caspase-1 (1:1000, Cell Signaling), anti-Emerin (1:2000, Abcam, Cambridge, UK), anti-HA (1:1000, Santa Cruz), and anti-GAPDH (1:10,000, Proteintech). Bound primary antibodies were detected using HRP-conjugated secondary antibodies (1:1000, Santa Cruz), and signals were visualized by chemiluminescence (Bio-Rad, Hercules, CA, USA). Densitometric analysis was performed using ImageJ (version 1.54p).

### 2.9. Co-Immunoprecipitation Assay

EA.hy926 cells were transfected as previously described with a SUMO-HA expression vector, either alone or in combination with pEGFP-C1-PML-IV. After 24 h, cells were stimulated with LPS (1 µg/mL) for 4 h, followed by ATP (5 mM) for 30 min, as indicated. Cells were lysed in ice-cold lysis buffer supplemented with protease inhibitors and 20 mM N-ethylmaleimide to preserve SUMO conjugation. Lysates were cleared by centrifugation and incubated with anti-HA magnetic beads (Thermo Fisher Scientific, USA) for 4 h at 4 °C with gentle rotation. Beads were then washed extensively using wash buffer provided by TBS-T buffer, and bound proteins were eluted with non-reducing SDS-Page sample buffer. Samples were resolved by SDS-PAGE and analyzed by immunoblotting using anti-NLRP3 antibodies. Input lysates were analyzed in parallel to verify expression and loading.

### 2.10. ELISA

IL-1β and IL-18 protein levels in cell culture supernatants were measured using a commercial human ELISA kit (Invitrogen by Thermo Fisher Scientific, Waltham, MA, USA) following the manufacturer’s instructions. Samples were analyzed in duplicates.

### 2.11. Immunofluorescence Analysis

Cellular localization of PML and ASC was analyzed in fixed EA.hy926 cells by immunocytochemistry followed by confocal laser scanning microscopy, as described previously [29]. After blocking with 10% FCS in PBS, cells were incubated overnight at 4 °C with anti-PML (1:50, Santa Cruz, Germany) or anti-ASC (1:100, Cell Signaling, USA) antibodies. Alexa Fluor–conjugated secondary antibodies (Life Technologies, Carlsbad, CA, USA) were applied in blocking buffer containing 5 µM Draq5 (nuclear marker) for 1 h at room temperature. After three PBS washes, samples were mounted with fluorescence mounting medium (Agilent, Santa Clara, CA, USA). Imaging was performed using a Leica DMI 6000 confocal microscope equipped with 20× and 63× oil-immersion objectives, and images were processed using Leica LAS AF Lite software.

### 2.12. Statistical Analysis

Data were analyzed using GraphPad Prism (version 10.6.1, San Diego, CA, USA) and are presented as mean ± SD from ≥3 independent experiments. Normality was tested using the Shapiro–Wilk test and variance homogeneity using the Brown–Forsythe test. Group comparisons were performed by one-way or two-way ANOVA with Tukey’s post hoc test. Statistical significance was set at *p* < 0.05.

## 3. Results

### 3.1. NLRP3 Inflammasome Activation in Endothelial Cells

**Figure 1:**
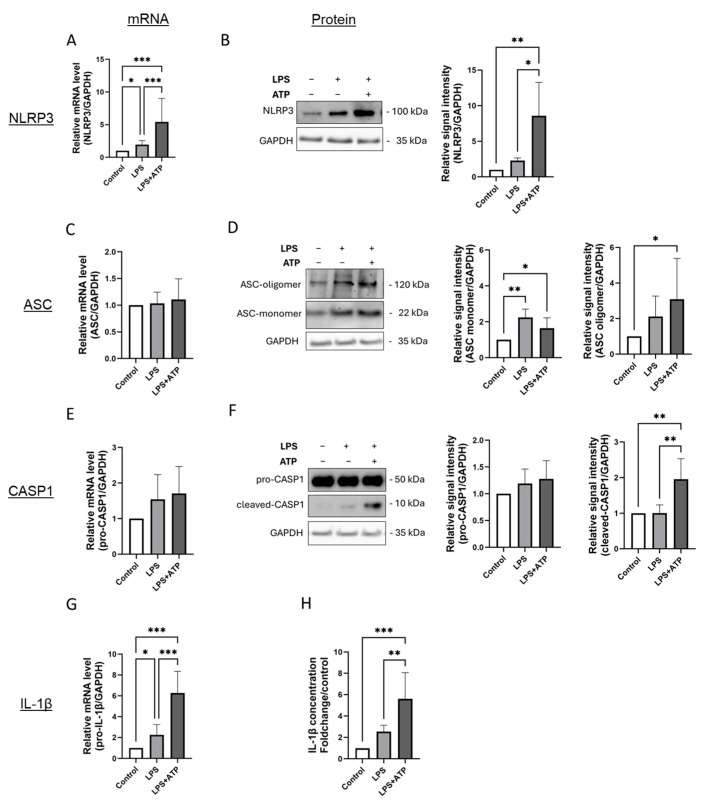
Inflammasome activation by LPS and ATP in EA.hy926 cells is characterized by higher expression of NLRP3, formation of ASC oligomers, higher activation of caspase-1 and more IL-1β expression and secretion. (**A**–**G**) mRNA and protein levels of inflammasome markers in EA.hy926 cells after incubation with LPS (1 µg/mL) for 24 h, with or without subsequent ATP (5 mM) stimulation for 1 h. mRNA and protein levels of NLRP3 (**A**,**B**), ASC (**C**,**D**), Caspase-1 (**E**,**F**), and IL-1β (**G**) were quantified using RT-qPCR or by immunoblotting of total protein lysates. Expression values are shown relative to untreated controls. *n* = 6, two-way ANOVA. Representative immunoblots from *n* = 6 experiments, two-way ANOVA. (**H**) Determination of IL-1β protein levels in supernatants of cultured EA.hy926 cells after incubation with LPS alone or LPS plus ATP. Expression values are shown relative to controls. *n* = 6 experiments, two-way ANOVA. All graphs are presented as mean ± SD. * *p* < 0.05, ** *p* < 0.01, *** *p* < 0.001.

To elucidate the role of PML in inflammasome activation in ECs, we first evaluated the cellular model system using LPS and ATP stimulation by analyzing the expression of specific inflammasome markers. Experiments were conducted using EA.hy926 cells (Figure 1) and HUVECs (Figure S1), with the monocyte cell line THP-1 (Figure S2) included to verify the inflammasome-activating responses observed in ECs and to facilitate comparison of the results.

Inflammasome activation was induced by priming the cells with 1 µg/mL LPS for 24 h, followed by ATP incubation at 5 mM concentration for 1 h prior to the end of the exposure period. To distinguish the effects of LPS alone, cells incubated without ATP were also analyzed.

In EA.hy926 cells, inflammasome activation following stimulation with LPS and ATP was evident by an increase in NLRP3 expression at both the mRNA and protein levels, formation of ASC oligomers, increased protein expression of cleaved-(active) caspase-1, as well as an upregulation of IL-1β gene expression accompanied by elevated IL-1β secretion (Figure 1A,B,D,F–H). Interestingly, LPS stimulation alone induced a rise in NLRP3 and IL-1β gene expression, which was further amplified upon inflammasome activation with ATP (Figure 1A,B,G,H). However, cleavage of caspase-1 was observed only in cells exposed to both LPS and ATP (Figure 1F). Inflammasome activation in EA.hy926 cells did not affect ASC mRNA levels (Figure 1C). At the protein level, ASC monomerand oligomer were constitutively expressed (Figure 1C). Protein expression of the ASC monomer was slightly higher in cells exposed to LPS and LPS plus ATP (Figure 1D). Inflammasome activation led to an approximately threefold higher formation of ASC oligomers compared to controls, although this was not significantly higher than after LPS stimulation alone (Figure 1D).

A similar activation pattern was observed in HUVECs (Figure S1). Notable differences were primarily detected in the mRNA expression of pro-caspase-1, which was significantly increased in HUVECs after combined LPS and ATP treatment compared to the control, but not significantly different from LPS stimulation alone (Figure S1E).

In THP-1 cells, similar to ECs, more cleaved caspase-1 and higher expression and secretion of IL-1β were observed after LPS and ATP stimulation (Figure S2G,H). Additionally, more oligomerization of ASC was evident in these cells, although in THP-1 cells, this was only seen after stimulation with LPS or LPS combined with ATP (Figure S2D). A similar expression pattern was observed for the presence of activated caspase in THP-1 cells (Figure S2F). Interestingly, stimulation of THP-1 cells did not lead to higher mRNA expression of NLRP3, although slightly elevated protein concentrations were observed, which were not higher than in cells stimulated with LPS alone (Figure S2A,B).

These findings provide a detailed characterization of inflammasome activation in cultured ECs, highlighting partially distinct responses in terms of NLRP3 expression and ASC oligomerization compared to those observed in cultured monocytes.

### 3.2. Priming Induces the Expression of PML and the Formation of PML-NBs in ECs

**Figure 2:**
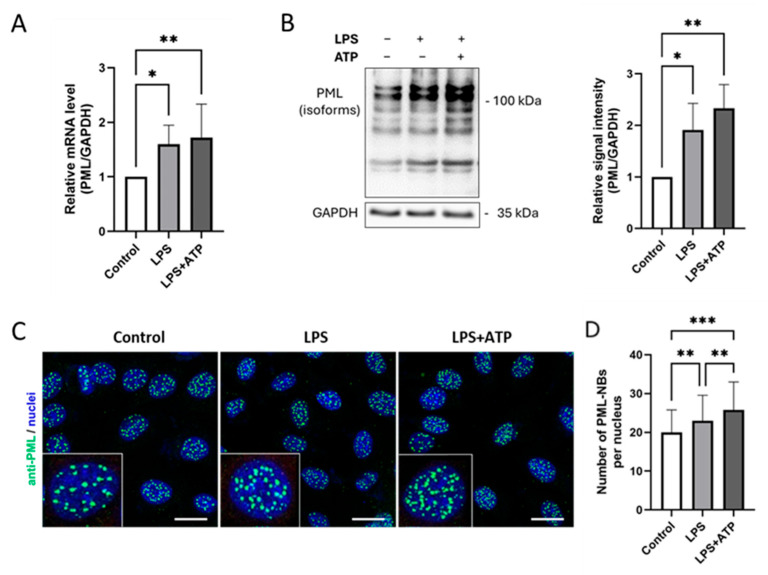
Inflammasome activation by LPS and ATP induces PML upregulation and more PML-NBs formation in EA.hy926 cells. (**A**,**B**) mRNA and protein levels of PML in EA.hy926 cells after incubation with LPS (1 µg/mL) for 24 h, with or without subsequent ATP (5 mM) stimulation for 1 h. Levels were quantified by RT-qPCR or immunoblotting of total protein lysates. mRNA values are shown relative to controls. *n* = 6, two-way ANOVA. Representative immunoblots from *n* = 4 experiments, two-way ANOVA. (**C**) Immunocytochemistry of EA.hy926 cells stained with anti-PML (green) and Draq5 for nuclei (blue). Representative images from *n* = 3 experiments. Scale bar: 20 µm. (**D**) The numbers of PML-NBs in nuclei of EA.hy926 incubated with LPS (1 µg/mL) for 24 h, with or without subsequent ATP (5 mM) stimulation for 1 h, were counted and compared to the numbers of PML-NBs in control cells. *n* = 60 from 3 independent experiments. All graphs are presented as mean ± SD. * *p* < 0.05, ** *p* < 0.01, *** *p* < 0.001.

To investigate whether the activation of the inflammasome influences the expression of PML and/or the formation and number of PML-NBs, EA.hy926 cells were stimulated with LPS or LPS plus ATP. Using the same samples as for the previous analyses, PML mRNA and protein levels were quantified by qPCR and immunoblotting and the localization and number of PML-NBs was determined by immunofluorescence (Figure 2).

The results show that PML mRNA expression was significantly higher after both LPS and LPS plus ATP stimulation (Figure 2A). Strikingly, additional ATP administration did not lead to a further significant increase in PML mRNA expression. This observation was also reflected at the protein level (Figure 2B). Interestingly, however, inflammasome activation led to a slight increase in the formation of additional PML-NBs, as visualized by immunofluorescence staining (Figure 2C). Similar results were observed in HUVECs (Figure S3A,B) and THP-1 cells (Figure S3C,D).

### 3.3. PML Increases the mRNA Levels of Inflammasome Components but Does Not Induce Inflammasome Activation

**Figure 3:**
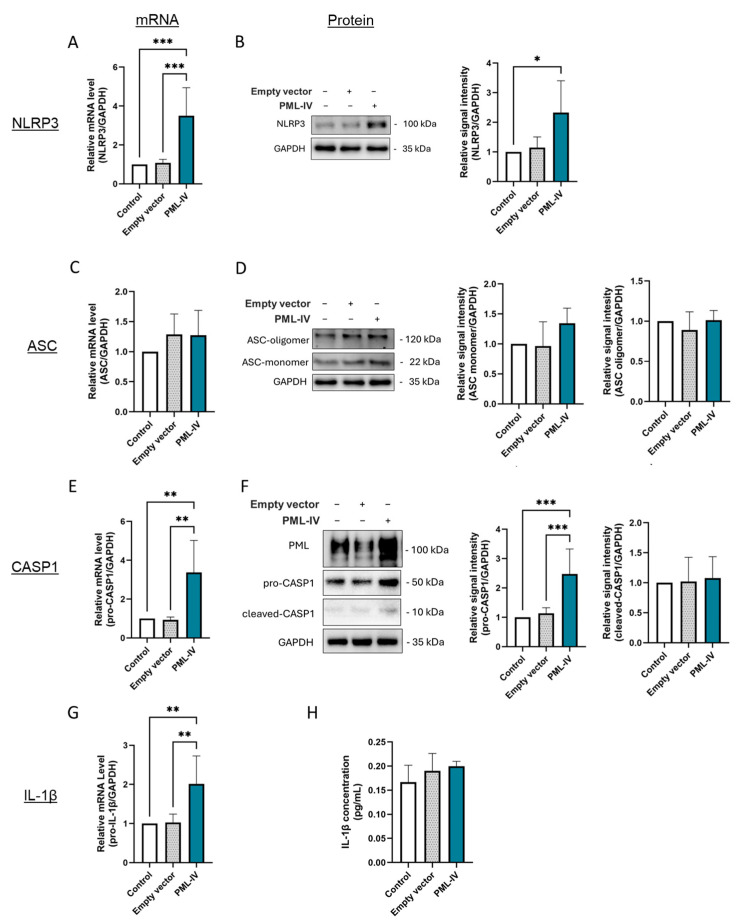
The effect of PML on LPS- and ATP-independent inflammasome activation in EA.hy926 cells. (**A**–**G**) mRNA and protein levels of inflammasome markers were measured in cells transfected with either an empty vector or pEGFP-C1-PML-IV (=PML-IV). mRNA and protein levels of NLRP3 (**A**,**B**), ASC (**C**,**D**), caspase-1 (**E**,**F**), and IL-1β (**G**) were quantified using RT-qPCR or immunoblotting of total protein lysates. Expression values are shown relative to controls (non-transfected cells). *n* = 6, one-way ANOVA. Representative immunoblots from *n* = 4–6 experiments, one-way ANOVA. (**H**) Determination of IL-1β protein levels in supernatants of EA.hy926 cells after transfection with a vector lacking a specific gene insert or a pEGFP-C1-PML-IV-vector. Expression values are shown relative to controls. *n* = 4 experiments, one-way ANOVA. All graphs are presented as mean ± SD. * *p* < 0.05, ** *p* < 0.01, *** *p* < 0.001.

To further characterize the influence of PML on inflammasome activation in ECs, EA.hy926 cells were transfected with a human PML-IV-specific vector (pEGFP-C1-PML- IV) (Figure 3 and Figure S4A,B). This resulted in higher mRNA and protein expression of NLRP3, pro-caspase-1 and increased mRNA levels of pro-IL-1β (Figure 3A,B,E,G), indicating a reinforcement of the inflammatory response. However, despite these changes, no induction of ASC oligomerization, augmented activation of caspase-1 or higher IL-1β secretion was observed (Figure 3D,F,H). This indicates that although increased PML expression in ECs influences the expression of inflammatory markers, it is not sufficient to initiate the formation and activation of the NLRP3 inflammasome.

### 3.4. PML Modulates NF-kB-Dependent Gene Expression of Inflammasome Markers in Endothelial Cells

**Figure 4:**
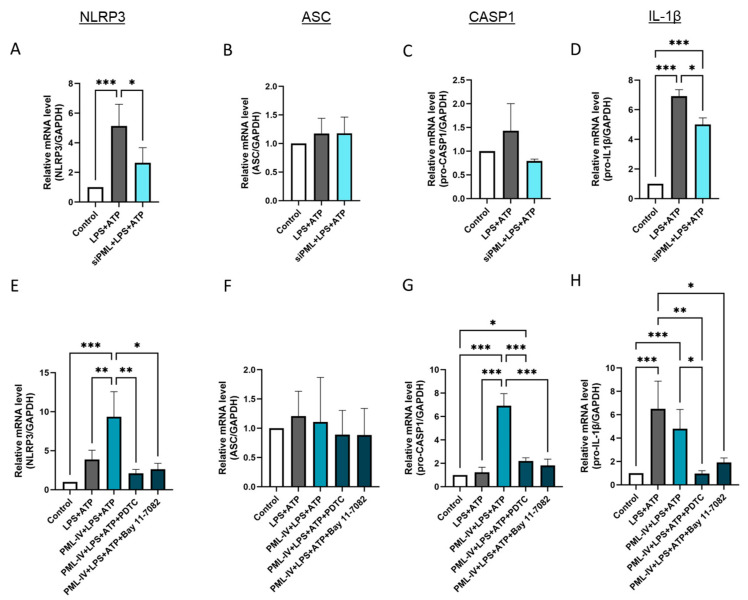
PML influences via NF-kB the mRNA levels of inflammasome markers after its activation by LPS and ATP in EA.hy926 cells. (**A**–**D**) The mRNA levels of inflammasome markers were determined in EA.hy926 cells after incubation with LPS (1 µg/mL) and ATP (5 mM) with or without transfection with PML-specific siRNAs (siPML). The mRNA levels of NLRP3 (**A**), ASC (**B**), pro-caspase-1 (**C**), and IL-1β (**D**) were quantified using RT-qPCR. Expression values are shown relative to controls. *n* = 3–4, two-way ANOVA. (**E**–**H**) The mRNA levels of inflammasome markers were determined in EA.hy926 cells after incubation with LPS (1 µg/mL) and ATP (5 mM) with or without transfection with the pEGFP-C1-PML-IV vector (=PML-IV) and additional PDTC (10 μM) or Bay 11-7082 (10 µM) treatment. The mRNA levels of NLRP3 (**E**), ASC (**F**), caspase-1 (**G**), and IL-1β (**H**) were quantified using RT-qPCR. Expression values are shown relative to controls. *n* = 7, two-way ANOVA. All graphs are presented as mean ± SD. * *p* < 0.05, ** *p* < 0.01, *** *p* < 0.001.

Next, the influence of PML on the gene expression levels of the markers NLRP3, ASC, caspase-1 and IL-1β during inflammasome activation was evaluated (Figure 4). After transfection with specific PML siRNA the inflammasome assembly and activation was induced by incubation with LPS and ATP in EA.hy926 cells, as previously described. The transfection efficiency was checked by RT-PCR and immunoblotting (Supplemental Figure S4C,D). The use of specific siRNA resulted in lower expression of NLRP3, caspase-1 and IL-1β in these cells compared to the non-transfected cells treated with LPS and ATP (Figure 4A,C,D). However, no effect on the mRNA levels of ASC was detected (Figure 4B). Overexpression of PML in combination with inflammasome activation led to higher NLRP3 and caspase-1 expression compared to cells treated with LPS and ATP alone, while no effect on ASC and IL-1β mRNA levels was noticed (Figure 4E–H). Notably, a modulatory effect of PML was not observed if the cells were treated with the NF-κB inhibitors PDTC or Bay 11-7082, as the mRNA levels of the markers corresponded to those in the untreated cells (Figure 4E,G,H). The results furthermore showed that IL-1β mRNA expression levels in EA.hy926 cells regulated by inflammasome activation were also NF-κB-dependent (Figure 4H).

Additionally, PML isoform 5 was tested at the mRNA level (Figure S5A–D). The effects tended to be similar to those of PML isoform IV, but markedly weaker.

To gain initial insight into whether PML could also influence the AIM2 inflammasome, PML-IV-overexpressing EA.hy926 cells were transfected with poly(dA:dT). No additional effect on AIM2 mRNA levels was observed compared to cells transfected with poly(dA:dT) alone (Figure S6).

### 3.5. PML Suppresses NLRP3 Inflammasome Assembly During Inflammasome Activation

**Figure 5:**
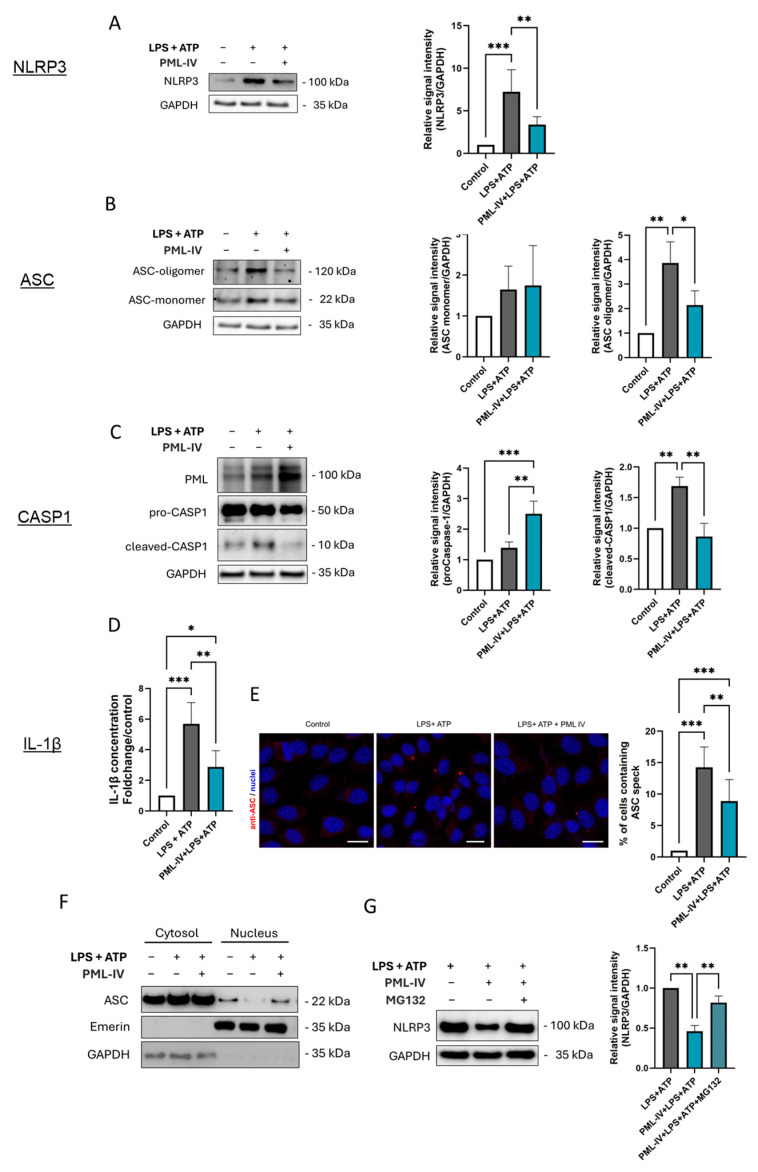
PML negatively influences inflammasome activation in EA.hy926 cells. (**A**–**D**) The protein levels of inflammasome markers were determined in EA.hy926 cells after incubation with LPS (1 µg/mL) and ATP (5 mM) with or without transfection with the pEGFP-C1-PML-IV vector (=PML-IV). The protein levels of NLRP3 (**A**), ASC (**B**), and caspase-1 (**C**), were quantified by immunoblotting. Expression values are shown relative to controls. Representative immunoblots from *n* = 4 experiments, two-way ANOVA. (**D**) Determination of IL-1β protein levels in supernatants of EA.hy926 cells after incubation with LPS and ATP alone with or without transfection with the pEGFP-C1-PML-IV vector. Expression values are shown relative to controls. *n* = 4 experiments, two-way ANOVA. (**E**) Immunocytochemistry using an anti-ASC (red) antibody on EA.hy926 cells treated as indicated. Draq5 staining was used for labeling of cell nuclei (blue). Representative images from *n* = 3 experiments. Scale bars 20 µm. (**F**) Cells were stimulated with LPS (1 µg/mL) and ATP (5 mM), with or without transfection of the pEGFP-C1-PML-IV vector. Nuclear and cytoplasmic fractions were analyzed by immunoblotting using antibodies against ASC, Emerin (nuclear marker), and GAPDH (cytoplasmic marker). Representative immunoblot of *n* = 3. (**G**) EA.hy926 cells were stimulated with LPS (1 µg/mL) and ATP (5 mM), with or without transfection of the pEGFP-C1-PML-IV vector, and additionally treated with the proteasome inhibitor MG132 (10 µM) or vehicle control. NLRP3 protein levels were quantified by immunoblotting. *n*  =  3. All graphs are presented as mean ± SD. * *p* < 0.05, ** *p* < 0.01, *** *p* < 0.001.

The effect of PML on the protein levels of the markers tested was then analyzed in more detail by PML transfection (Figure 5). Interestingly, higher NLRP3 protein levels and more activation of caspase-1 was not observed (Figure 5A,C). On the contrary, overexpression of PML even led to a lower protein concentration of NLRP3 and active caspase-1 after exposure of the cells to LPS and ATP. The formation of ASC oligomers and the secretion of IL-1β were also lower after the additional overexpression of PML isoform IV compared to inflammasome activation without plasmid transfection (Figure 5B,D). Consistent with these findings, PML knockdown experiments confirmed that, following inflammasome activation, PML-depleted cells exhibited increased NLRP3 protein levels as well as enhanced secretion of IL-1β and IL-18 (Figure S7).

To investigate the temporal dynamics of PML’s inhibitory effect on inflammasome activity, additional time points following ATP stimulation were examined, including 15 min, 30 min, and 2 h (Figure S8). The inhibitory effect of PML on ASC oligomerization was already evident at 15 min and persisted throughout the 120 min observation period. A slight decrease in IL-1β secretion was observed after 30 min, was clearly pronounced after one hour (Figure 5D) and was still detectable after two hours.

In addition, immunofluorescence staining was employed to visualize ASC specks, a hallmark of inflammasome assembly, in order to investigate the extent to which NLRP3 inflammasome formation is regulated by PML. Consistent with our other findings, the number of ASC specks was reduced in PML-overexpressing and stimulated cells compared to cells stimulated with LPS and ATP alone (Figure 5E).

Given that PML has been shown to influence the subcellular localization of ASC in macrophages, we extended this analysis to ECs (Figure 5F). Upon stimulation, ASC was more strongly retained in the nucleus when PML was overexpressed.

To determine whether the PML-mediated reduction of NLRP3 is proteasome-dependent, cells were additionally treated with the proteasome inhibitor MG132. While PML overexpression markedly reduced NLRP3 protein levels, MG132 treatment restored NLRP3 levels nearly to those of the corresponding non-transfected cells, indicating that PML promotes proteasomal degradation of NLRP3 (Figure 5G). In addition, cycloheximide (CHX) chase experiments were performed to directly assess protein turnover (Figure S9). In PML-overexpressing cells, NLRP3 degradation occurred significantly faster, with a marked reduction already detectable 15 min after CHX treatment, followed by a plateau, whereas in non-transfected control cells, a substantial decrease was only observed at the 3 h time point.

### 3.6. The Formation of Functional PML Nuclear Bodies Is Crucial for the Inhibition of NLRP3 Inflammasome Assembly in Endothelial Cells

**Figure 6:**
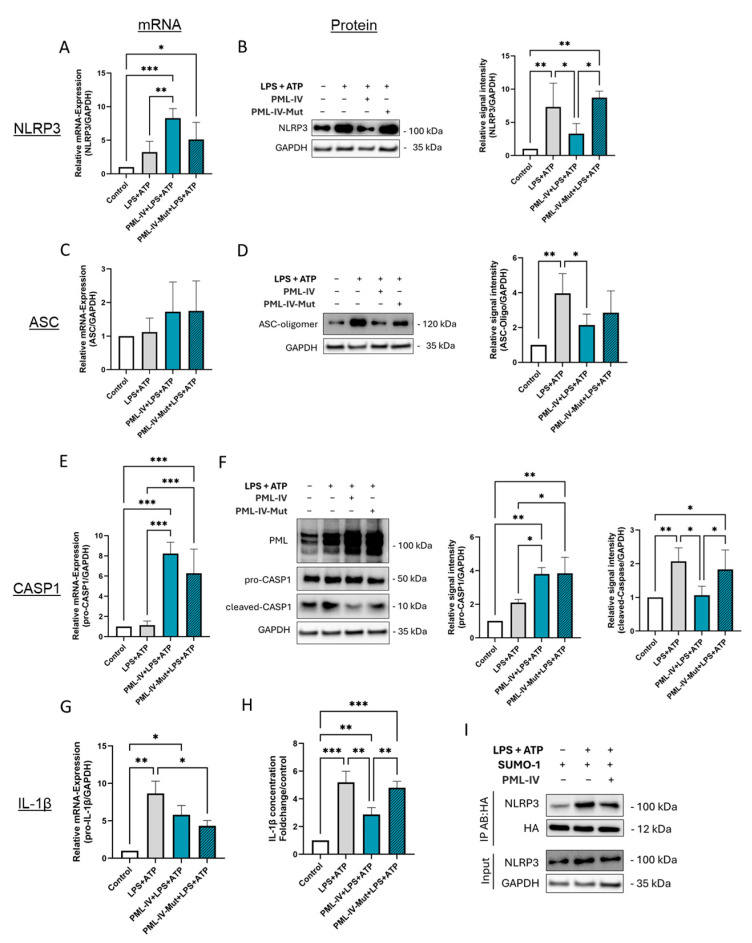
SUMOylation of PML is obligatory to inhibit inflammasome activation of EA.hy926 cells. (**A**–**G**) mRNA and protein levels of inflammasome markers in EA.hy926 cells after incubation with LPS (1 µg/mL) and ATP (5 mM) with or without transfection with the wild-type PML-IV vector (=PML-IV) or a SUMO-defective PML-IV vector (=PML-IV-Mut). mRNA and protein levels of NLRP3 (**A**,**B**), ASC (**C**,**D**), caspase-1 (**E**,**F**), and IL-1β (**G**) were quantified using RT-qPCR or immunoblotting of total protein lysates. Expression values are shown relative to controls. *n* = 4–6, two-way ANOVA. Representative immunoblots from *n* = 3–4 experiments, two-way ANOVA. (**H**) Determination of IL-1β protein levels in supernatants of EA.hy926 cells after incubation with LPS (1 µg/mL) and ATP (5 mM) with or without transfection with the pEGFP-C1-PML-IV vector or a SUMO-defective PML-IV vector. Expression values are shown relative to controls. *n* = 4 experiments, two-way ANOVA. (**I**) EA.hy926 cells were transfected with pcDNA3-HA-SUMO1 expression vector, either alone or in combination with pEGFP-C1-PML-IV, and subsequently treated with LPS (1 µg/mL) and ATP (5 mM) as indicated. Cell lysates were subjected to immunoprecipitation using anti-HA antibodies to isolate SUMOylated proteins. Imunmunoprecipitates were analyzed by immunoblotting for NLRP3 to assess its SUMOylation status. *n* = 3. All graphs are presented as mean ± SD. * *p* < 0.05, ** *p* < 0.01, *** *p* < 0.001.

To assess the influence of the SUMOylation state and consequently the formation of properly assembled PML-NBs, EA.hy926 cells were transfected either with a mutated PML-IV construct (PML-3/pSG5), encoding a PML-IV protein carrying three lysine-to-alanine substitutions within the SUMOylation consensus motifs (K65, K160, K490) and thereby lacking SUMOylation activity, or with wild-type PML-IV (pEGFP-C1-PML-IV) (Figure 6).

Overexpression of the SUMOylation-deficient mutant had effects on the mRNA expression of NLRP3, caspase-1, and IL-1β, comparable to those induced by wild-type PML. However, no corresponding reductions were observed at the protein level (Figure 6A,B,E–H). Notably, inflammasome assembly and activation were not suppressed by the mutant, as evidenced by unchanged levels of NLRP3 expression, ASC oligomer formation, and IL-1β expression and secretion compared to controls (Figure 6B,D,H). Given the central role of PML-NBs in regulating post-translational modifications (PTMs) such as SUMOylation, we next investigated whether NLRP3 is SUMOylated in ECs and to what extent PML modulates this process. Immunoprecipitation experiments demonstrated that NLRP3 is indeed SUMOylated in ECs, with SUMOylation levels increasing upon stimulation with LPS and ATP (Figure 6I). Interestingly, co-transfection with wild-type PML resulted in reduced SUMOylation of NLRP3 compared to the total amount of NLRP3 detected in the IP eluate (Figure 6I).

In summary, the results indicate that PML suppresses inflammasome formation upon activation by reducing NLRP3 protein levels, impairing ASC oligomerization, downregulating active caspase-1, and decreasing IL-1β secretion.

## 4. Discussion

**Figure 7:**
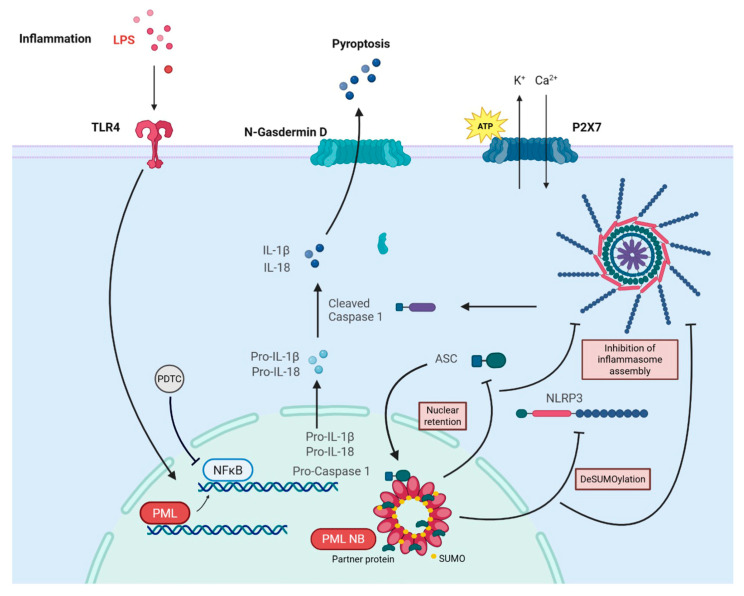
Schematic summary of the molecular model proposed in this study. In response to LPS priming and ATP-induced NLRP3 inflammasome activation, PML exerts a dual function: (1) it enhances NF-κB-dependent transcription of inflammasome components such as NLRP3 and pro-IL-1β, and (2) it simultaneously restrains inflammasome assembly by promoting nuclear retention of ASC and modifying NLRP3 post-translationally. This leads to lower NLRP3 protein levels, diminished ASC oligomerization, leading to limited caspase-1 activation and IL-1β secretion.

The results of this study provide new insights into the role of PML in the regulation of inflammasome activation in ECs, as summarized schematically in Figure 7. We found a PML-dependent induction of the transcriptional rates of key NF-kB-dependent inflammasome components, such as NLRP3, caspase-1 and IL-1β, in the absence of fully assembled and activated inflammasomes. Instead, PML appeared to act as a negative regulator of inflammasome assembly, limiting the downstream activation processes required for caspase-1 cleavage and IL-1β secretion.

Inflammasome assembly and activation is a well-studied process in macrophages and other immune cells [30]. In contrast, this process has been much less studied in ECs, particularly in EA.hy926 cells, although LPS and ATP are known to trigger inflammasome activation in these cells [31,32]. Therefore, the monocyte cell line THP-1 was included to validate and compare the cellular responses observed in ECs. Our results confirm that LPS and ATP stimulation trigger hallmark features of NLRP3 inflammasome activation in ECs (Eahy.926 and HUVECs), including higher NLRP3 expression, ASC oligomerization, caspase-1 cleavage, and IL-1β secretion. Of note, in contrast to THP-1 cells, we observed a certain degree of basal inflammasome activity in non-stimulated ECs, which is consistent with previous reports [33,34] and indicates an intrinsic low-level inflammatory state in cultured ECs. IL-1β levels also differed between THP-1 monocytes and cultured ECs. At 0.01 pg/mL to a maximum of 7.52 pg/mL, IL-1β levels were significantly lower in ECs than in monocytes. The low IL1β secretion by ECs, especially by EA.hy926, has already been described by Abas et al. [35,36,37]. In summary, our results show that ECs are capable of triggering an inflammasome response, albeit one that is much less pronounced than that of classical immune cells.

Inflammasome activation in ECs led not only to the upregulation of classical inflammasome markers but also increased the expression of the PML protein, which is known to contribute to the formation of nuclear multiprotein aggregates known as PML-NBs during inflammatory responses [25]. An elevated number of PML-NBs was observed even upon stimulation with LPS alone, suggesting that priming is sufficient to enhance PML expression in ECs. This is consistent with earlier findings that proinflammatory cytokines such as interferons [38] and IL-6 [39,40] promote PML expression and PML-NB formation. In ECs, PML has also been shown to be inducible by TNF-α and interferons α and γ [41,42]. However, a direct link between LPS stimulation and PML gene regulation in ECs has not previously been established.

To date, the role of PML in inflammasome signaling has been examined exclusively in macrophages, yielding conflicting results. While one study reported a promoting effect of PML on NLRP3 inflammasome activation [43], others suggested an inhibitory role [44,45]. Whether PML exerts a similar modulatory effect on inflammasome activity in ECs has thus far remained unclear.

A striking observation in this study was the dissociation between inflammasome gene expression and protein-level activation in the context of PML protein modulation. While PML upregulated mRNA levels of inflammasome-related genes, it failed to induce downstream activation events such caspase-1 cleavage, or IL-1β secretion. To explore the underlying mechanism, we first investigated whether PML acts via NF-κB. PML knockdown reduced NLRP3 and pro-caspase-1 transcript levels, and overexpression enhanced their expression following LPS and ATP stimulation. These effects were reversed by the NF-κB inhibitors PDTC or Bay 11-7082, implicating NF-κB as a mediator of PML-dependent gene regulation. Interestingly, pro-IL-1β mRNA levels remained unaffected by PML overexpression, likely due to cell type-specific expression limits in EA.hy926 and HUVECs. The sustained pro-caspase-1 expression despite NF-κB inhibition points to additional, NF-κB-independent regulatory mechanisms. Associations between PML and NF-κB with controversial effects have already been published [46,47]. The present study indicates an activating influence of PML on NF-κB.

As mentioned before, overexpression of PML in ECs induced transcription of several NLRP3 inflammasome components, but did not lead to higher protein expression or assembly of the inflammasome. On the contrary, NLRP3 protein levels, ASC oligomer formation, caspase-1 activation, and IL-1β secretion were markedly reduced compared to control cells. This was also supported by immunofluorescence imaging showing diminished ASC speck formation, indicating impaired inflammasome assembly. A similar inhibitory role of PML in inflammasome activation has been described in macrophages, where PML1 was shown to colocalize with ASC, sequestering it in the nucleus and thereby preventing inflammasome activation [45]. In resting cells, ASC predominantly resides in the nucleus and translocates to the cytosol upon inflammasome activation [45,48]. To test whether a similar mechanism operates in ECs, subcellular fractionation was performed. Significantly higher levels of nuclear ASC were detected in PML-overexpressing ECs compared to controls, indicating PML-mediated retention of ASC in the nucleus.

Mechanistically, our data therefore suggest that PML impairs inflammasome assembly at multiple levels. First, PML enhances NF-κB-dependent transcription of inflammasome components and primes cells for activation. Second, PML promotes nuclear retention of ASC, likely preventing its cytosolic oligomerization and subsequent signaling. Third, despite higher mRNA levels, NLRP3 protein expression was reduced, suggesting post-transcriptional destabilization. Our results indicate that, under inflammatory conditions, the reduction in NLRP3 is proteasome-dependent: treatment with the proteasome inhibitor MG132 restored NLRP3 protein levels in PML-overexpressing cells to those of non-transfected controls, and cycloheximide chase experiments demonstrated accelerated NLRP3 degradation in PML-overexpressing cells. The rapid decline of NLRP3 within 15 min in PML-IV overexpressing cells, followed by a plateau, may be consistent with a SUMO-dependent, proteasome-mediated degradation mechanism. This suggests that only a subset of NLRP3, potentially the SUMOylatable pool, is targeted for rapid degradation, while the remaining protein remains relatively stable.

Given the established role of PML-NBs in coordinating PTMs such as SUMOylation and ubiquitination, we hypothesized that PML-NBs modulates NLRP3 stability through PTM-dependent mechanisms.

Supporting this notion, overexpression of a SUMOylation-deficient PML mutant—which is thought to lack structurally intact PML-NBs due to its inability to interact properly with partner proteins [49]—failed to suppress inflammasome assembly or reduce NLRP3 protein levels, despite inducing gene expression changes comparable to those triggered by wild-type PML. This indicates that the presence of functionally intact PML-NBs is essential for mediating the observed effects on NLRP3 regulation. Since SUMOylation represents a hallmark function of PML-NBs and NLRP3 is known to undergo SUMOylation [50,51,52]—although the functional implications of these modifications remain controversial—we initially focused on investigating the SUMOylation status of NLRP3.

Our co-immunoprecipitation analyses revealed that NLRP3 undergoes increased SUMO1 modification in ECs upon inflammasome activation, consistent with previous findings by Shao et al. [52]. Interestingly, PML overexpression showed reduced NLRP3- SUMOylation, suggesting that PML may act as a negative regulator by limiting SUMO1 conjugation following priming. This effect could result from interference with the SUMOylation machinery or enhanced de-SUMOylation by SUMO-specific proteases (SENPs), which are known to localize within PML-NBs. Reduced SUMOylation may, in turn, facilitate proteasomal degradation of NLRP3 by exposing ubiquitination sites—a mechanism previously proposed by Qin et al. [51] and supported by our data. Collectively, these findings suggest that PML modulates SUMOylation dynamics within PML-NBs to promote NLRP3 degradation, thereby limiting inflammasome activity in ECs.

The ability of PML to limit inflammasome activation in ECs emphasizes its potential role in protecting against excessive endothelial inflammation. This regulatory mechanism may be particularly relevant in vascular pathologies such as atherosclerosis and sepsis, where dysregulated inflammasome activity and endothelial dysfunction are key contributors to disease progression [6,11,53]. Notably, increased expression of both NLRP3 and PML has been observed in atherosclerotic lesions [54,55], pointing to a complex interplay between pro- and anti-inflammatory signaling pathways.

By restricting inflammasome assembly, PML may prevent maladaptive inflammatory responses, thereby protecting against tissue damage and chronic vascular inflammation. Our findings therefore highlight a previously underappreciated role of PML in maintaining endothelial homeostasis under inflammatory conditions.

One of the main limitations of this study is that all experiments were conducted exclusively in vitro using cell culture models. While these models are valuable for dissecting cellular mechanisms, they do not fully replicate the complex interactions and microenvironments present in a living organism.

Another limiting factor of this work is that overexpression analyses focused primarily on PML Isoform IV. Although a few experiments were performed with PML Isoform V, another nuclear isoform that showed similar but less pronounced effects, it remains possible that other PML isoforms may differentially influence inflammasome activation.

Therefore, the potential roles of additional isoforms should be addressed in future studies.

Furthermore, inflammasome activation was induced using only one specific combination of pathophysiological relevant stimuli—LPS and ATP. Other relevant modulators, such as heme and shear stress, which are also known to influence inflammasome assembly in ECs [56,57], were not examined.

Although an initial experiment indicated that PML does not affect AIM2 mRNA levels, further studies are needed to clarify whether PML also influences the activity of other inflammasomes, such as AIM2 or NLRC4, in addition to NLRP3.

Another aspect that warrants exploration in more depth is the investigation of other PTMs, such as phosphorylation or ubiquitination, which could play a role in regulating inflammasome activation and the stability of NLRP3 and ASC.

Finally, future studies should further assess the influence of PML on inflammasome activation in specific disease-relevant cell and animal models to better understand the clinical relevance of these findings in different disease contexts such as atherosclerosis, sepsis or viral infections.

## 5. Conclusions

In summary, this study identifies PML as a critical modulator of inflammasome activation in ECs. PML promotes transcription of inflammasome components via NF-κB, but simultaneously inhibits inflammasome assembly and activation by sequestering ASC in the nucleus and regulating NLRP3 protein levels presumably via SUMOylation-dependent mechanisms. These findings extend our understanding of PML’s immunomodulatory functions and highlight its potential relevance in vascular inflammatory diseases.

## Figures and Tables

**Table 1:** Primer sequences, product sizes and annealing temperatures used to amplify the corresponding cDNA templates.

Template	Forward Primer	Reverse Primer	Product Size	Annealing Temperature
**PML**	5′-CCG CAA GAC CAA CAA CAT CTT-3′	5′-CAG CGG CTT GGA ACA TCC T-3′	91 bp	58 °C
**Pro-CASP1**	5′-TGC CCA CAG ACA TTC ATA CAG TTT C-3	5′-GCC TGT TCC TGT GAT GTG GAG-3′	166 bp	60 °C
**ASC**	5′-GCC TGC ACT TTA TAG AC-3′	5′-GCT TCC GCA TCT TGC TTG G-3′	153 bp	52 °C
**NLRP3**	5′-CCA CAA GAT CGT GAG AAA ACC C C-3′	5′-CGG TCC TAT GTG CTC GTC A-3′	91 bp	58 °C
**Pro-IL1β**	5′-CAC GAT GCA CCT GTA CGA TCA-3′	5′-GTT GCT CCA TAT CCT GTC CCT-3′	121 bp	58 °C
**GAPDH**	5′-ATG ACC TTG CCC ACA GCC TT-3′	5′-AAC TGC TTA GCA CCC CTG GC-3′	200 bp	60 °C

## Data Availability

All data generated or analyzed during this study are included in this published article.

## Supplementary Materials

The following supporting information can be downloaded at: https://www.mdpi.com/article/10.3390/cells14241961/s1, Figure S1: Inflammasome activation in HUVECs; Figure S2: Inflammasome activation in THP1 cells; Figure S3: Inflammasome activation leads to higher PML expression in HUVECs and THP1 cells; Figure S4: Verification of transfection efficiency in EA.hy926 cells by RT-qPCR and immunoblotting; Figure S5: Influence of PML isoform V on the mRNA expression of inflammasome markers in EA.hy926 cells; Figure S6: Effect of PML-IV overexpression on AIM2 mRNA expression in EA.hy926 cells under inflammatory conditions; Figure S7: Impact of PML-specific siRNA on NLRP3 protein levels and IL-1β/IL-18 secretion in EA.hy926 cells following LPS and ATP stimulation; Figure S8: Time-dependent effects of PML-IV overexpression on ASC oligomerization and IL-1β secretion in EA.hy926 cells under inflammatory conditions; Figure S9: PML-IV overexpression modulates NLRP3 protein stability in EA.hy926 cells under inflammatory conditions.

## Abbreviations

The following abbreviations are used in this manuscript:

ASC	apoptosis-associated speck-like protein containing a CARD
ATP	adenosine triphosphate
CHX	Cycloheximide
DMSO	dimethyl sulfoxide
ECs	endothelial cells
HUVEC	human umbilical vein endothelial cells
LPS	Lipopolysaccharide
NLRP3	NOD-like receptor pyrin domain-containing 3
PAMPs	pathogen-associated molecular patterns
PDTC	Pyrrolidine dithiocarbamate
PML	promyelocytic leukemia protein
PML-NBs	PML nuclear bodies
PRRs	pattern-recognition receptors
PTMs	post-translational modifications
ROS	reactive oxygen species
SENPs	Sentrin/SUMO-specific proteases
SUMO	small ubiquitin-related modifier
