# Supplementary material for: PML Is Limiting NLRP3 Inflammasome Activity in Human Endothelial Cells
Source: Cells. 2025 Dec 10;14(24):1961. doi: 10.3390/cells14241961 (PMC12732286; doi:10.3390/cells14241961)
Supplement: Supplementary file 1 [file cells-14-01961-s001.zip › cells-3891338-supplementary.pdf]

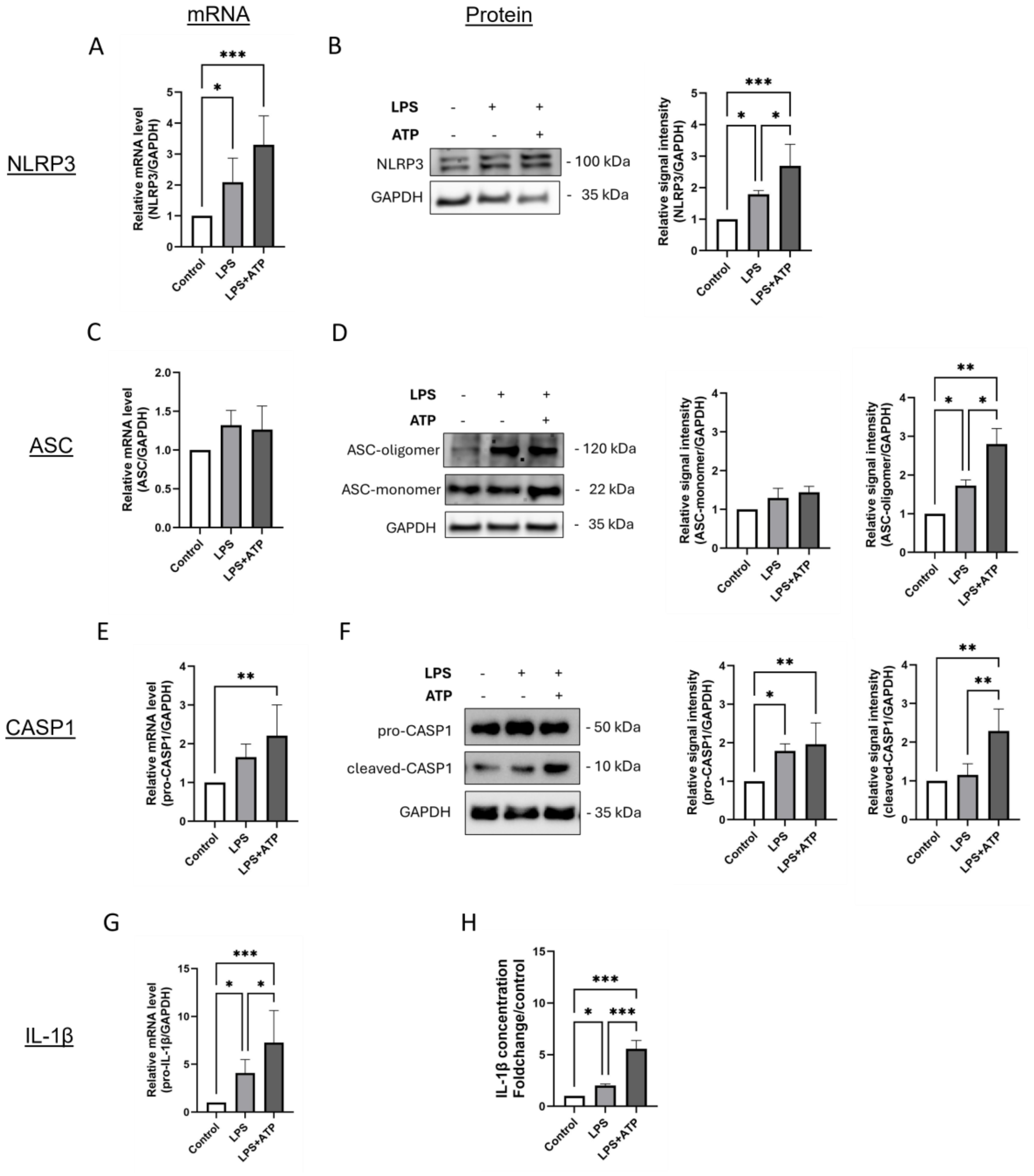

Supplemental Figure S1. Inflammasome activation in HUVECs. (A-G) mRNA and protein levels of inflammasome markers in HUVECs after incubation with LPS (1 µg/ml) for 24 h, with or without subsequent ATP (5 mM) stimulation for 1 h. mRNA and protein levels of NLRP3 (A, B), ASC (C, D), caspase-1 (E, F), and IL-1β (G) were quantified using RT-qPCR or immunoblotting of total protein lysates. Expression values are shown relative to controls. n = 6, two-way ANOVA. Representative immunoblots from n = 3-5 experiments, two-way ANOVA. (H) Determination of IL-1β protein levels in supernatants of HUVECs cells after incubation with LPS alone or LPS plus ATP. Expression values are shown relative to controls. n = 4 experiments, two-way ANOVA. All graphs are presented as mean ± SD. *$p < 0.05$, **$p < 0.01$, ***$p < 0.001$.

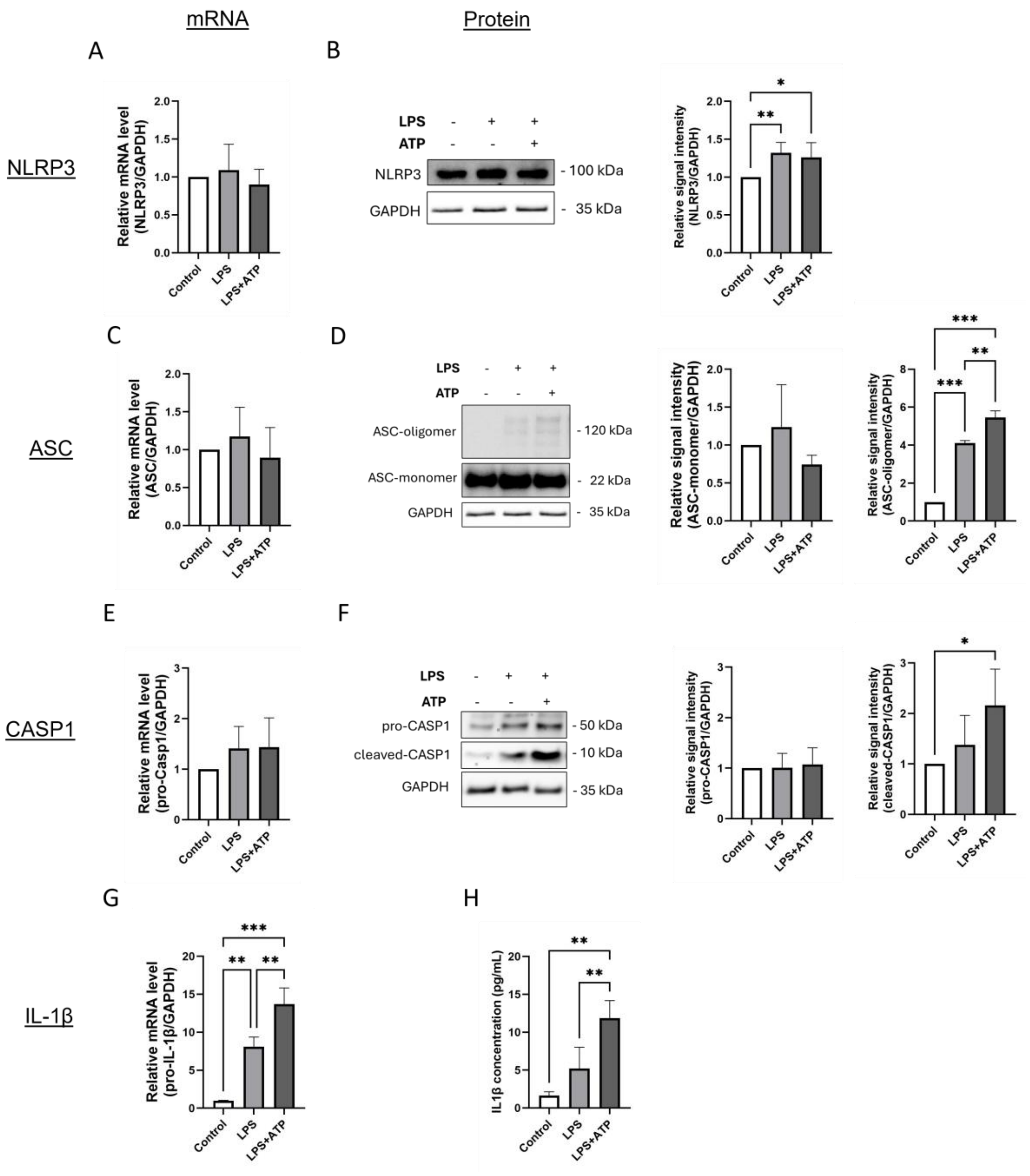

Supplemental Figure S2. Inflammasome activation in THP1 cells. (A-G) mRNA and protein levels of inflammasome markers in THP1 cells after incubation with LPS (1 μg/ml) for 24 h, with or without subsequent ATP (5 mM) stimulation for 1 h. mRNA and protein levels of NLRP3 (A, B), ASC (C, D), caspase-1 (E, F), and IL-1β (G) were quantified using RT-qPCR or immunoblotting of total protein lysates. Expression values are shown relative to controls. n = 8, two-way ANOVA. Representative immunoblots from n = 4-6 experiments, two-way ANOVA. (H) Determination of IL-1β protein levels in supernatants of THP1 cells after incubation with LPS alone or LPS plus ATP. Expression values are shown relative to controls. n = 4 experiments, two-way ANOVA. All graphs are presented as mean ± SD. *$p < 0.05$, **$p < 0.01$, ***$p < 0.001$.

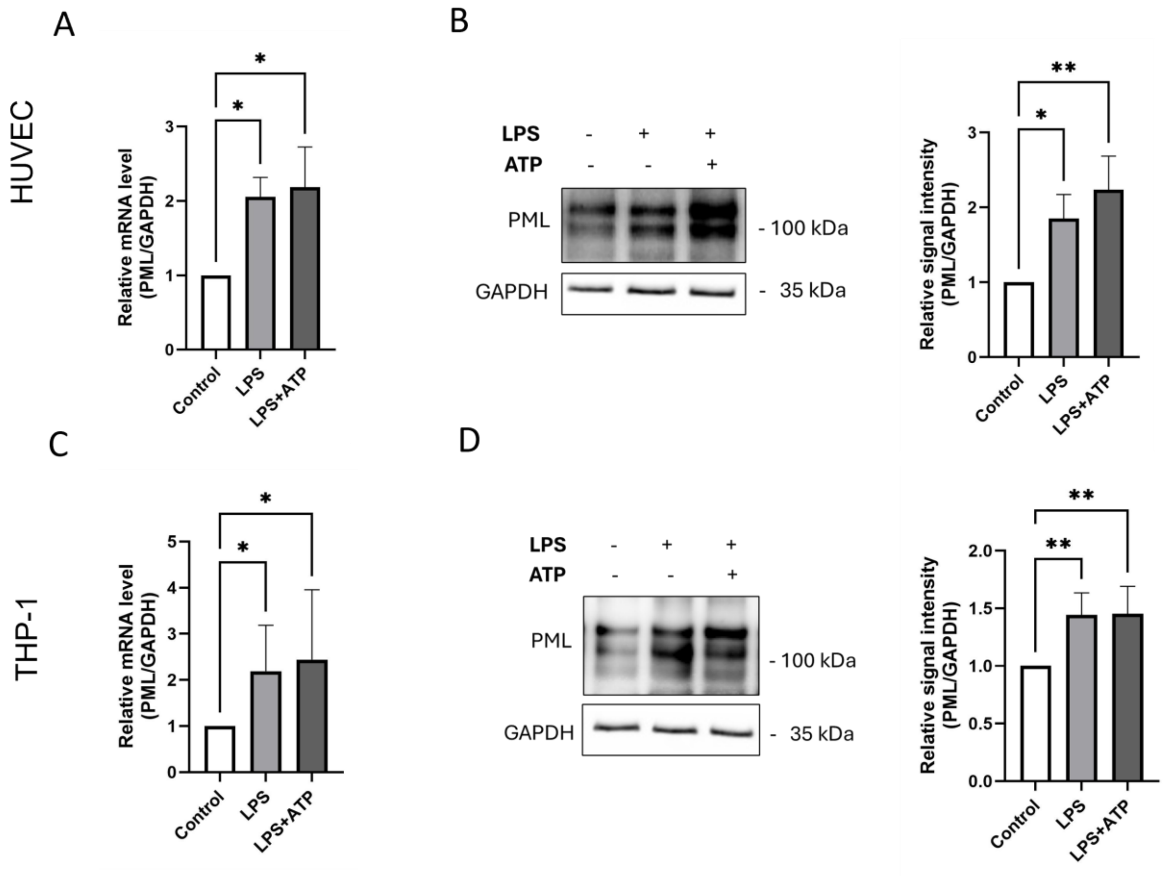

Supplemental Figure S3. Inflammasome activation leads to higher PML expression in HUVECs and THP1 cells. (A-D) mRNA and protein levels of PML in HUVECs and THP1 cells after incubation with LPS (1 µg/ml) for 24 h, with or without subsequent ATP (5 mM) stimulation for 1 h. mRNA and protein levels PML in HUVECs (A, B) or Thp1 cells (C, D), were quantified using RT-qPCR (A, C) or by immunoblotting (B, D) of total protein lysates. Expression values are shown relative to controls. n = 4/8, two-way ANOVA. Representative immunoblots from n = 4/5 experiments, two-way ANOVA. All graphs are presented as mean ± SD. *$p < 0.05$, **$p < 0.01$, ***$p < 0.001$.

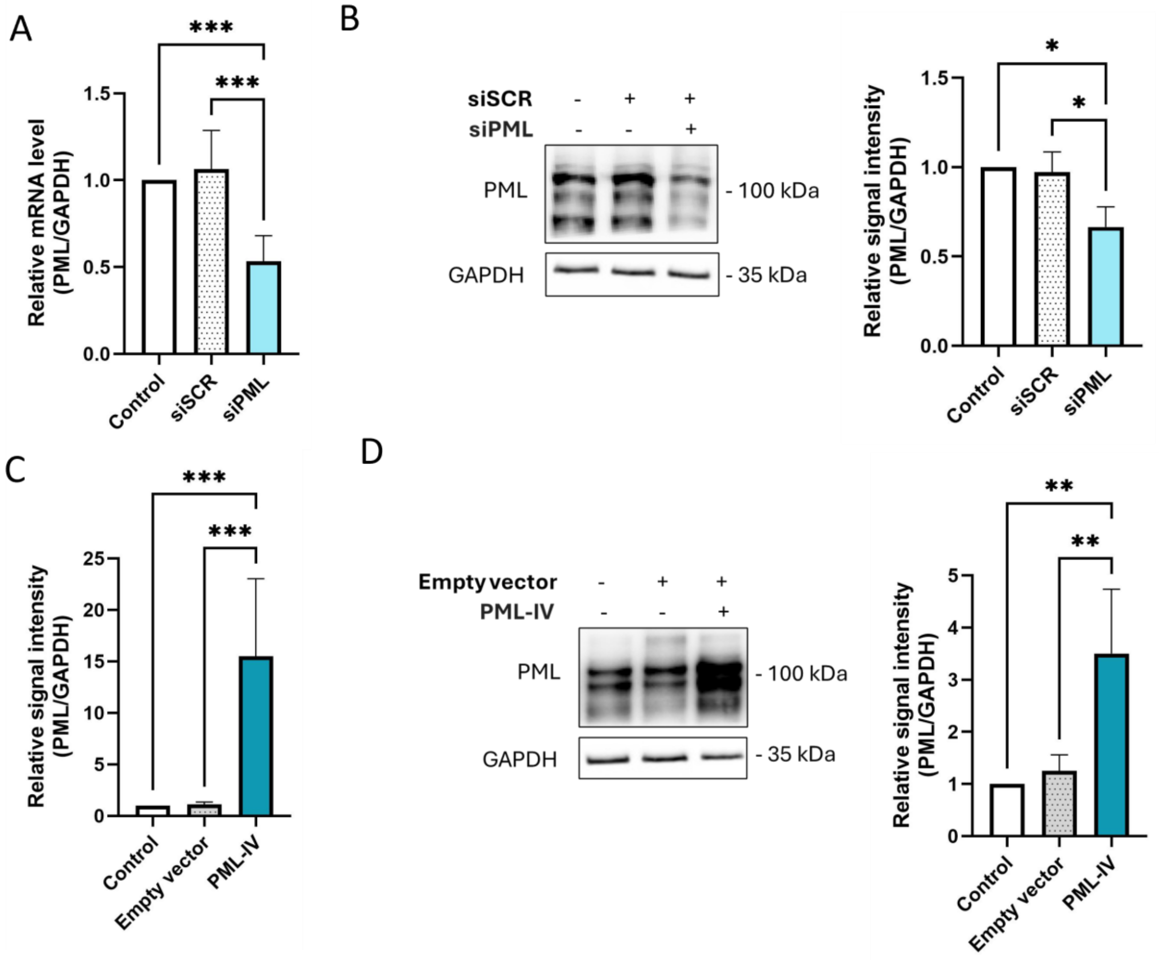

Supplemental Figure S4. Verification of transfection efficiency in EA.hy926 cells by RT-qPCR and immunoblotting. mRNA and protein levels of PML in EA.hy926 cells after transfection with PML-specific or scrambled siRNAs (A, B) or the pEGFP-C1-PML-IV (=PML-IV) or the corresponding empty vector (C, D) were quantified by RT-qPCR (A, C) and immunoblotting of total protein lysates (B, D). Expression values are shown relative to controls. n = 6, one-way ANOVA. Representative immunoblots from n = 3 experiments, one-way ANOVA. All graphs are presented as mean ± SD. *p < 0.05, **p < 0.01, ***p < 0.001.

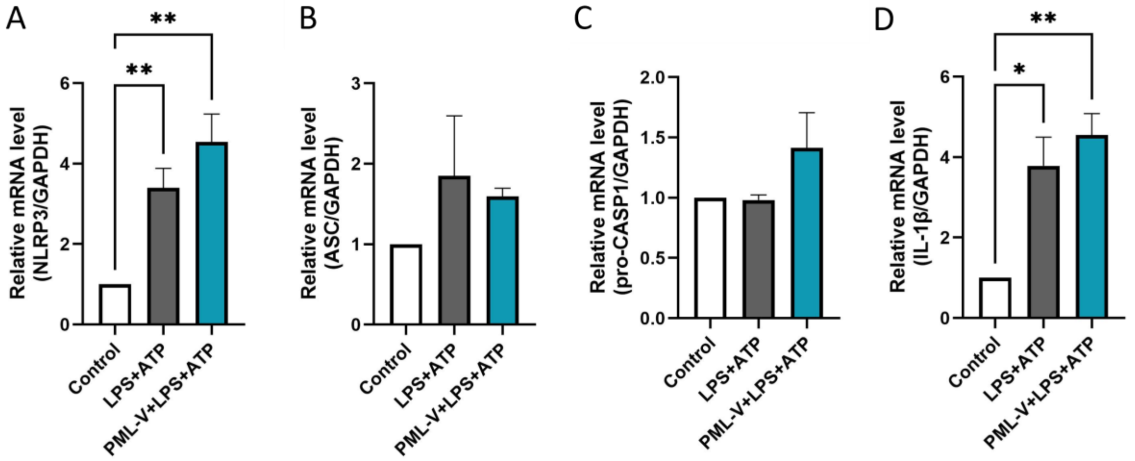

Supplemental Figure S5. Influence of PML isoform V on the mRNA expression of inflammasome markers in EA.hy926 cells. (A-D) The mRNA levels of inflammasome markers were determined in EA.hy926 cells after incubation with LPS (1 µg/ml) and ATP (5 mM) with or without transfection with the pEGFP-C1-PML-V vector (= PML-V). The mRNA levels of NLRP3 (A), ASC (B), caspase-1 (C), and IL-1β (D) were quantified using RT-qPCR. Expression values are shown relative to controls. n = 3, two-way ANOVA. All graphs are presented as mean ± SD. *p < 0.05, **p < 0.01.

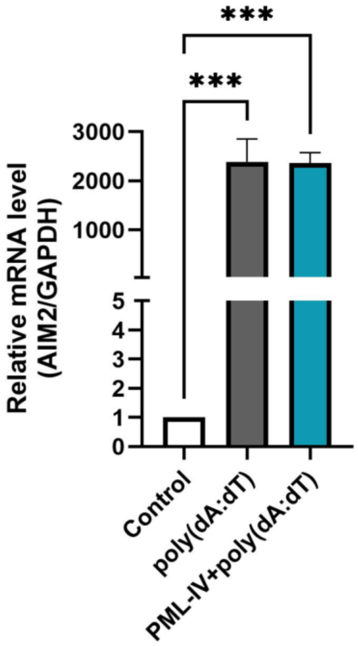

Supplemental Figure S6. Effect of PML-IV overexpression on AIM2 mRNA expression in EA.hy926 cells under inflammatory conditions. The mRNA levels of AIM2 were measured in EA.hy926 cells with or without transfection of poly(dA:dT) (5 µg/ml) and with or without PML-IV overexpression. The mRNA levels of AIM2 were quantified using RT-qPCR. Expression values are shown relative to unstimulated controls. n = 3, two-way ANOVA. All graphs are presented as mean ± SD. ***p < 0.001.

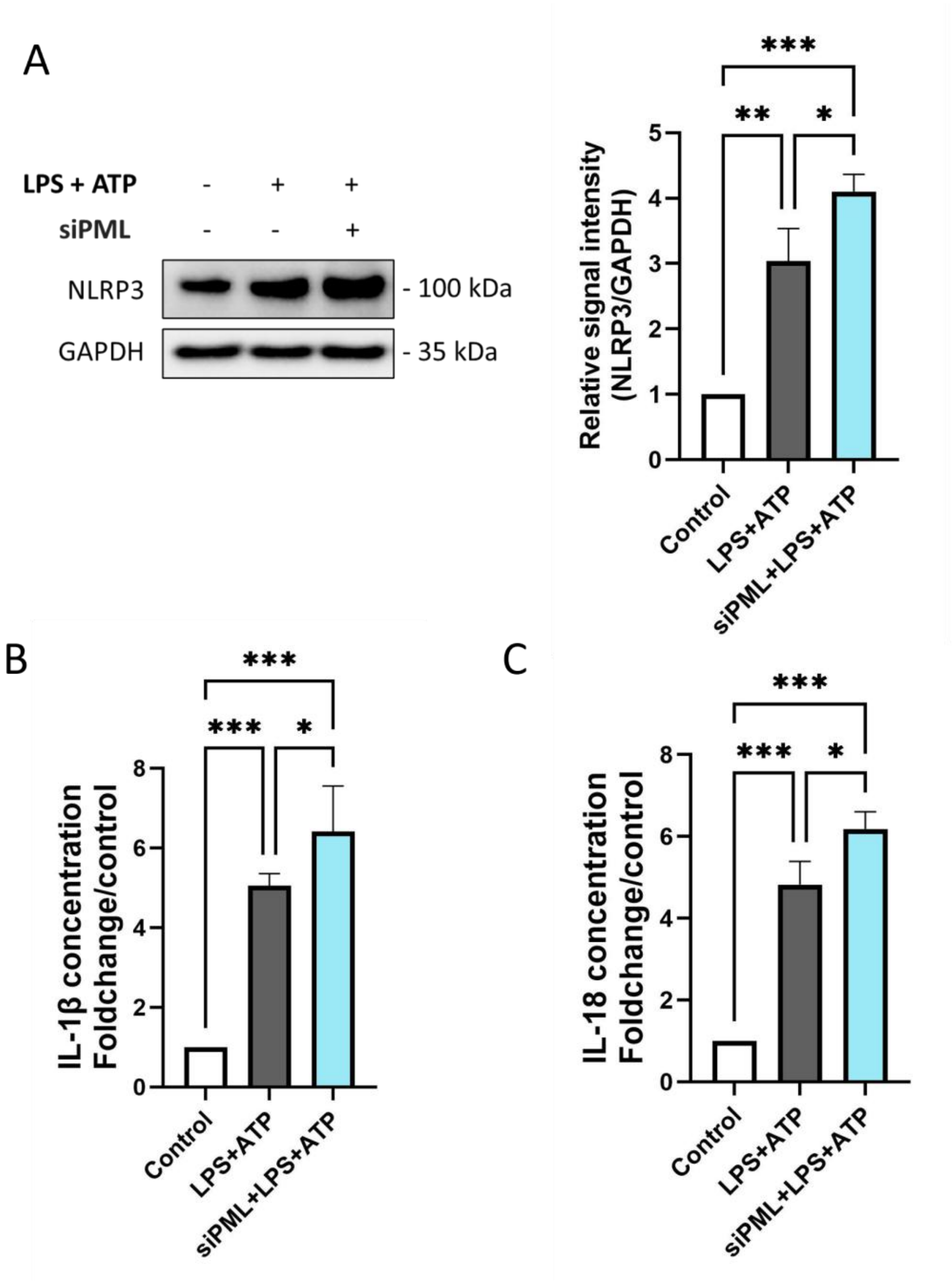

Supplemental Figure S7. Impact of PML-specific siRNA on NLRP3 protein levels and IL-1β/IL-18 secretion in EA.hy926 cells following LPS and ATP stimulation. (A–C) The protein levels of inflammasome markers were determined in EA.hy926 cells after incubation with LPS (1 μg/ml) and ATP (5 mM) with or without transfection with PML-specific siRNAs (siPML). (A) The protein levels of NLRP3 were quantified by immunoblotting. Expression values are shown relative to controls. Representative immunoblots from n = 3 experiments, two-way ANOVA. (B,C) Determination of IL-1β (B) and IL-18 (C) protein levels in supernatants of EA.hy926 cells were determined by ELISA. Expression values are shown relative to controls. n = 4 experiments, two-way ANOVA. All graphs are presented as mean ± SD. *p < 0.05, **p < 0.01, ***p < 0.001.

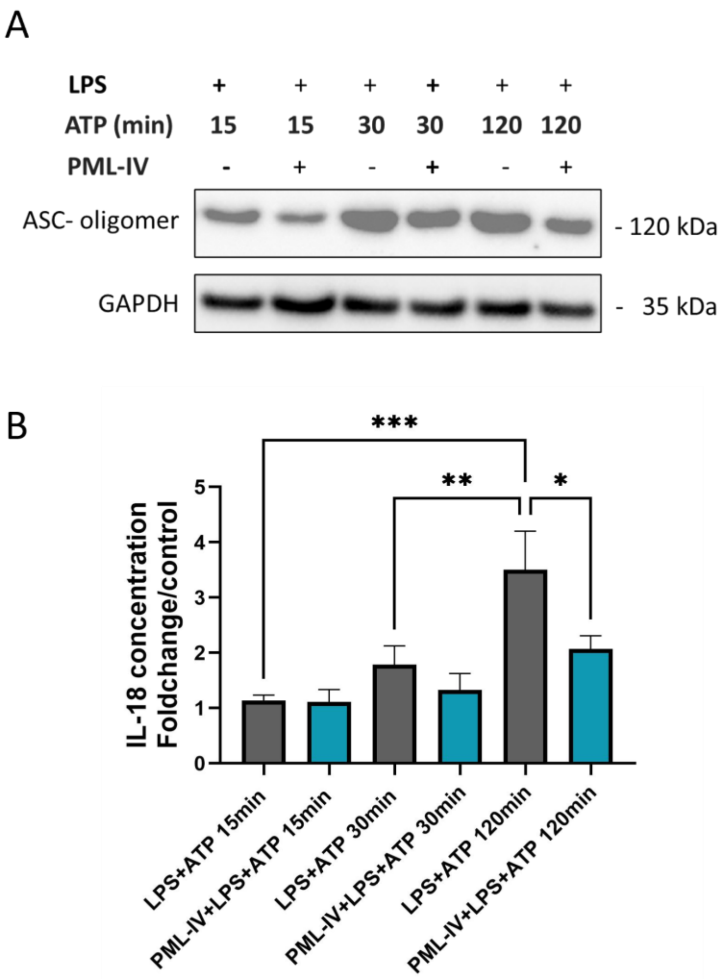

Supplemental Figure S8. Time-dependent effects of PML-IV overexpression on ASC oligomerization and IL-1β secretion in EA.hy926 cells under inflammatory conditions. (A,B) To analyze the kinetics of ASC oligomerization, EA.hy926 cells were stimulated with LPS (1 μg/ml) followed by ATP (5 mM) for the indicated time points, with or without transfection of the pEGFP-C1-PML-IV vector (PML-IV). ASC oligomer levels were quantified by immunoblotting. (B) IL-1β protein levels in the cell culture supernatants were quantified by ELISA under the same stimulation conditions. Expression values are shown relative to unstimulated control cells. n = 3 experiments, two-way ANOVA. All graphs are presented as mean ± SD. *p < 0.05, **p < 0.01, ***p < 0.001.

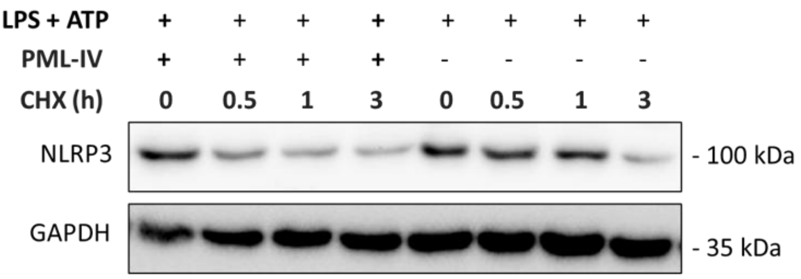

Supplemental Figure S9. PML-IV overexpression modulates NLRP3 protein stability in EA.hy926 cells under inflammatory conditions. EA.hy926 cells were incubated with LPS (1 µg/ml) and ATP (5 mM), with or without transfection of the pEGFP-C1-PML-IV vector (PML-IV), and treated with the translation inhibitor cycloheximide (CHX; 10µg/ml) for the indicated time points. NLRP3 protein levels were quantified by immunoblotting.
